# The associations between alcohol intake and cardiometabolic risk in African-origin adults spanning the epidemiologic transition

**DOI:** 10.1186/s12889-021-12128-2

**Published:** 2021-12-04

**Authors:** Danny Baghdan, Lara R. Dugas, Candice Choo-Kang, Jacob Plange-Rhule, Pascal Bovet, Bharathi Viswanathan, Terrence Forrester, Estelle V. Lambert, Walter Riesen, Wolfgang Korte, Mashkoor A. Choudhry, Amy Luke

**Affiliations:** 1 Parkinson School of Health Sciences & Public Health, Loyola University Chicago, 2160 S. 1st Ave, Maywood, IL 60153 USA; 2 Division of Epidemiology and Biostatics, School of Public Health & Family Medicine, University of Cape Town, Cape Town, South Africa; 3 Kwame Nkrumah University of Science and Technology, Kumasi, Ghana; 4 University of Lausanne, Lausanne, Switzerland; 5 Ministry of Health and Social Development, Public Health Authority, Victoria Hospital, Mahé, Republic of Seychelles; 6 Solutions for Developing Countries, University of the West Indies, Mona, Kingston, Jamaica; 7 Research Unit for Exercise Science and Sports Medicine, University of Cape Town, Cape Town, South Africa; 8 Center for Laboratory Medicine, Canton Hospital, St. Gallen, Switzerland; 9 Stritch School of Medicine, Loyola University Chicago, Maywood, IL USA

**Keywords:** African-origin populations, Cardiometabolic risk, Alcohol consumption, And Epidemiologic transition

## Abstract

**Background:**

Cardiometabolic (CM) risk affects approximately 25% of adults globally, and is diagnosed by meeting 3 out of 5 of the following CM risk factors: elevated blood pressure, high triglycerides, elevated blood sugar, low high-density lipoprotein (HDL) level, and abdominal obesity. Adults with CM risk are approximately 22% more likely to have higher mortality rates, and alcohol consumption may be associated with higher CM risk. While previous studies have investigated this potential connection, the majority of them did not include African-origin adults. Therefore, the study aimed to explore the association between alcohol intake and CM risk in 5 African-origin cohorts, spanning the epidemiologic transition in Ghana, South Africa, Jamaica, Seychelles and the United States of America.

**Methods:**

Measurements included clinical measures for CM risk and self-reported alcohol consumption. Each participant was categorized into one of three drinking categories: non-drinker, light drinker (1–3 drinks daily for men and 1–2 drinks daily for women) and heavy drinker (4 or more drinks every day for men and 3 or more drinks per day for women). Using non-drinker status as the reference, the association between alcohol consumption status and prevalence of each of the five CM risk factors and overall elevated CM risk (having 3 out of 5 risk factors) was explored, adjusting for site, age and sex. Associations were explored using logistic regression and significance was determined using odds ratios (OR) and 95% confidence intervals.

**Results:**

Neither light nor heavy drinking was associated with increased odds for having higher CM risk compared to nondrinkers (OR = 1.05, *p* = 0.792 and OR = 1.11, *p* = 0.489, respectively). However, light drinking was associated with lower odds for having low high density lipoproteins (HDL) cholesterol (OR = 0.69, *p* = 0.002) and increased risk for high triglycerides (OR = 1.48, *p* = 0.030). Heavy drinking was associated with elevated blood pressure (OR = 1.59, *p* = 0.002), high triglycerides (OR = 1.73, *p* = 0.006) and decreased risk of low HDL-cholesterol (OR = 0.621, *p* < 0.0005). Finally, country-specific analyses indicated that the relationship between heavy drinking and elevated CM risk varied widely across sites.

**Conclusion:**

While several CM risk factors were associated with alcohol consumption, the associations were inconsistent and varied widely across five international cohorts of African-origin. Future studies should focus on understanding the individual site-related effects.

**Supplementary Information:**

The online version contains supplementary material available at 10.1186/s12889-021-12128-2.

## Background

Cardiometabolic (CM) risk affects approximately 25% of adults globally [1, 2]. The National Cholesterol Education Program Expert Panel on Detection, Evaluation and Treatment of High Blood Cholesterol in Adults defines diagnosis as having any three of the following five CM risk factors: elevated blood pressure, high triglycerides (TG), elevated blood sugar, low high-density lipoprotein (HDL) level and abdominal obesity [3–5]. It is estimated that mortality rates are approximately 22% higher for adults with high CM risk compared to healthy adults [6]. While several non-modifiable risk factors, including age and sex have been determined to increase CM risk; the contribution of alcohol consumption remains inconsistent [7–10]. For example, many studies have demonstrated that the amount of alcohol intake is associated with individual CM risk factors; including hypertension, hypertriglyceridemia and hyperglycemia [7–9, 11, 12]. The level of alcohol consumption may show either increased or decreased risk for the individual CM factors, [13–17]. Despite these independent associations, data are lacking showing a causal link between alcohol consumption and overall CM risk. Given the international prevalence of alcohol consumption, with some countries reporting 80% of the population drinking regularly, and the global burden of high CM risk, this potential association warrants additional examination [1, 2, 18–20].

Recently, a meta-analysis from the Global Burden of Disease Study group examined 694 studies from 195 countries between 1990 and 2016, and found that alcohol consumption significantly increased CM risk [10]. This contradicts previous studies, which found that low levels of alcohol consumption may be protective against CM risk [21, 22]. For example, Freiberg et al. [22], reported that adults who regularly drink small amounts of alcohol have 40% lower odds for increased CM risk than adults who do not consume alcohol. However, this effect was less significant in African American participants than in white participants. The contradictory findings between these two studies suggests that any proposed associations have not yet been confirmed.

Previous studies have shown genetic/phenotypic relationships between country of origin and CM health. Factors such as abdominal obesity, HDL-cholesterol and other measures of CM health vary greatly due to differences in dietary and behavioral behaviors among countries [23, 24]. For example, a study that examined CM risk in 195 countries showed that the prevalence of high CM risk is higher in developing urban communities than in rural or fully developed areas, suggesting an epidemiologic effect on CM risk [23]. This may be as a result, in part, due to a higher caloric intake and other behavioral factors associated with urban populations as well as a higher prevalence of obesity in the latter than the former. A study examining rural and urban populations from 200 countries found obesity to be associated directly and fairly strongly with higher HDL-cholesterol, triglycerides and blood sugar levels [25].

Some studies have examined CM risk in African-origin populations living in Africa. However, to our knowledge, no previous studies have examined the relationship between alcohol consumption and CM risk have included African-origin participants living in Africa. Previous studies have already shown that different populations are not similarly affected by the separate factors, and that each population may respond to alcohol consumption differently [13–15, 26]. Therefore, the aim of this investigation was to examine the association between alcohol consumption and elevated CM risk in adults of African-origin using data from the Modeling the Epidemiologic Transition study (METS) [13, 14].

## Methods

METS is a prospective cohort study examining physical activity and weight change in 5 African-origin cohorts from Ghana, Jamaica, South Africa, Seychelles, and the United States of America (USA) (*N* = 500 at each site). METS was initiated in 2009. In total, 2506 participants of African origin, aged 25–45 years were recruited. Approximately 50% of participants were women (*N* = 1339), and all participants self-identified as African-origin. Sites were categorized using the 2010 United Nations’ Human Development Index (HDI) [15]. In 2010, Ghana was ranked as low HDI (0.554), South Africa was ranked as moderate (0.645), Seychelles and Jamaica were ranked as high (0.747 and 0.712, respectively), and the USA was ranked as very high (0.914). Data for the current study were collected during the baseline clinic visit, between the months of January 2009 and December 2011. The protocol for METS was approved by the Institutional Review Board of Loyola University Chicago, IL, USA (LU#200038); the Committee on Human Research Publication and Ethics of Kwame Nkrumah University of Science and Technology, Kumasi, Ghana; the Research Ethics Committee of the University of Cape Town, South Africa; the Board for Ethics and Clinical Research of the University of Lausanne, Switzerland; the Research and Ethics Committee, Ministry of Health and Social Development, Seychelles Public Health Department, Republic of Seychelles, and the Ethics Committee of the University of the West Indies, Kingston, Jamaica. A written informed consent was obtained from all participants before enrollment.

Study inclusion criteria included participants who self-identified as African-origin and considered themselves healthy. Participants with obvious infectious disease, HIV positive individuals, women who were either lactating or pregnant, and participants unable to engage in normal physical activity were not eligible for the study due to the impact these conditions had on CM risk.

METS participants presented annually for a health check, which included CM risk measurements, and self-reported alcohol consumption. Two alcohol consumption metrics were used to estimate drinking behavior: the number of drinks per day and the frequency of drinking. The clinical measurements were used to estimate the prevalence of CM risk at each of the five sites.

### Clinical measurements

All measurements were collected at outpatient research clinics in the respective communities. Weight (kg) and height (m) were collected while participants wore light clothing and no shoes, using identical procedures and equipment models across all five sites. This information was used to calculate body mass index (BMI) (kg/m^2^). BMI was used both continuously and categorically as “not overweight” (BMI < 25 kg/m^2^) and “overweight/obese (BMI ≥ 25 kg/m^2^). Waist circumference was also collected using tape measures and recorded in both centimeters and inches.

#### Blood pressure

Blood pressure (BP) measurements were performed by trained personnel using an automatic digital monitor (Omron model HEM-747Ic). Measurements were taken with the antecubital fossa at heart level. BP was measured twice during the research visit with at least an hour in between measurements. BP was measured in triplicate each time, thus resulting in a total of six BP measurements. BP was coded to be a dichotomous variable based upon whether the participant was hypertensive. In concordance with the American Heart Association’s definition, elevated blood pressure was identified by systolic blood pressure ≥ 130 mmHg, diastolic blood pressure ≥ 90 mmHg or if the participant was being treated for high blood pressure [26].

### Biochemical measures

Blood glucose levels (mg/dL), cholesterol (mg/dL) and triglyceride levels (mg/L) were obtained during the clinic examination. Participants were asked to fast 10–12 h prior to examination, during which fasting blood glucose was measured and blood samples were drawn by venipuncture using standard procedure. Fasting capillary glucose concentrations were determined using finger stick (Accu-check Aviva, Roche). Blood samples were briefly processed, and plasma or serum separated within 2 h of collection. Afterwards, all samples were stored in the laboratory at − 80 °C. Elevated blood glucose was defined as ≥100 mg/dL or receiving treatment for type 2 diabetes, while elevated high triglyceride levels were defined as ≥ 150 mg/dL or receiving treatment [3, 4]. Low HDL cholesterol was defined as < 40 mg/dL in men and < 50 mg/dL in women or receiving treatment [3, 4].

### Physical activity

All participants wore an accelerometer (Phillips Respironics, Bend, OR, USA), and instructed to wear it continuously over their right hip. They were advised to only remove it before bathing, showering, or swimming as the device was not waterproof. The monitor measures and records vertical accelerations to represent the intensity of each movement. The intensity and duration of physical activity was summarized in one minute epochs over the course of seven days. Light, moderate and vigorous intensity activity were defined as < 1535 counts per minute (cpm), 1535–3959 cpm and ≥ 3960 cpm, respectively [27]. Physical activity was measured as minutes performed per day.

### Questionnaires

All questionnaires were administered by trained personnel, as previously described [14]. The questionnaires inquired on each participant’s health information with a focus on cardiovascular disease and type 2 diabetes. Data collected included dietary habits, medication use, physical activity behavior and age of diagnosis for relevant illnesses. The study also collected data on household characteristics, participant occupation, education, parental education, household assets and amenities. Questions were based on the Core Welfare Indicators Questionnaire from the World Bank [28].

### Drinking status

The main exposure for the current study was alcoholic intake, and participants were asked about their alcohol consumption during the staff-administered interviews, and the responses were used to determine patterns of alcohol consumption. The Alcohol Use Disorder Identification Test was used to determine daily alcohol consumption, which includes questions such as: “In the past 12 months, how often have you drink (in days)? Per Week, Per Month, Per Year?”, “On those days that you drank, how many drinks did you have?”, and “On special occasions, how many drinks do you have?” Participants were categorized into 3 alcohol consumption groups, using the guidelines provided by the National Institute of Alcohol Abuse and Alcoholism (NIAAA) [29]. These categories were non-drinkers (who indicated no alcohol consumption), light drinker (who consumed 1–3 drinks daily for men and 1–2 drinks daily for women) and heavy drinker (who had 4 or more drinks every day for men and 3 or more drinks per day for women).

### Cardiometabolic risk

The main goal of the study was to measure elevated CM risk. Participants were classified with high CM risk, if they met any three of the following five CM criteria established by the Adult Treatment Panel III [3, 4]. These included abdominal obesity (waist circumference > 102 cm in males and > 88 cm in females); elevated BP (≥130/80 mmHg), or receiving treatment for hypertension; high TG (≥150 mg/dL); low HDL (HDL cholesterol < 40 mg/dL in males and < 50 mg/dL in females); and elevated blood glucose (fasting plasma glucose ≥100  mg/dL).

### Statistical analyses

Participant demographic and clinical data were summarized using mean ± standard deviation, both individually and overall. Categorical variables were displayed using N and frequency. The relationship between alcohol consumption (no alcohol, light drinking or heavy drinking) and CM risk was explored using logistic regression, and final results were determined using odds ratio (OR) and 95% confidence intervals (CI). Both daily consumption and drinking frequency were used for these comparisons. Abstaining from alcohol consumption was used as the reference category to which light and heavy drinkers were compared. All models were adjusted for age, sex, smoking status (smoker or non-smoker), self-reported physical activity and site (dummy variables). Statistical analyses were performed using Stata (version 12, Manufacturer, College Station, TX, USA) (year 2011). An alpha *p*-value of 0.05 was used to denote statistical significance.

## Results

### Participant characteristics

The descriptive characteristics of the five cohorts are presented in Tables 1 and 2, by site and sex. For men, the mean age ranged from 33.7 ± 5.8 years in South Africa to 36.4 ± 5.1 years in the Seychelles (Table 1). For women, the mean age ranged from 33.1 ± 6.0 years in South Africa, to 35.8 ± 5.6 years in the Seychelles (Table 2). Overall, participants from Ghana had the lowest BMI and the subjects from the USA had the highest BMI (Tables 1 and 2).

**Table 1:** Demographic data, by site for men

	Ghana	South Africa	Jamaica	Seychelles	United States	Overall
**N**	207	236	249	230	245	1167
**Mean Age**	34.6 ± 6.7	33.7 ± 5.6**	34.0 ± 5.9**	36.4 ± 5.1	35.6 ± 6.2	34.9 ± 6.0
**Weight (Kg)**	63.6 ± 9.1**	65.6 ± 13.6**	73.1 ± 15.0**	80.1 ± 16.0**	92.7 ± 24.8	75.4 ± 19.8
**Height (cm)**	169.0 ± 6.6**	170.9 ± 6.3**	176.0 ± 6.7	173.9 ± 6.2**	176.6 ± 6.6	173.4 ± 7.1
**BMI (Kg/m2)**	22.2 ± 2.7**	22.4 ± 4.3**	23.6 ± 4.5**	26.5 ± 4.9**	29.7 ± 7.5	25.0 ± 5.8
**Waist (cm)**	77.2 ± 10.4**	81.0 ± 11.5**	80.3 ± 12.1**	89.4 ± 11.8**	97.2 ± 21.6	85.2 ± 16.0
**Hip (cm)**	91.7 ± 10.9**	94.6 ± 8.4**	95.1 ± 9.3 **	102.8 ± 9.6**	109.2 ± 15.9	98.9 ± 12.9
**WHR**	0.84 ± 0.054**	0.85 ± 0.058**	0.84 ± 0.059**	0.87 ± 0.059*	0.88 ± 0.090	0.86 ± 0.067
**Overweight/Obese, (N) %**	31 (14.98)**	48 (20.34)**	74 (29.72)**	134 (58.26)**	176 (71.02)	532 (45.7)
**Smokers, (N) %**	13 (6.28)**	152 (64.41)	106 (42.91)**	86 (37.39)**	175 (71.72)	697 (27.79)
**MVPA Duration (min/day)**	46.9 ± 24.6**	55.5 ± 34.6**	29.6 ± 23.3	35.9 ± 24.3	33.1 ± 34.7	40.1 ± 30.6

*p < 0.05, and **p < 0.01. Reference is USA

**Table 2:** Demographic data, by site for women

	Ghana	South Africa	Jamaica	Seychelles	United States	Overall
**N**	293	268	251	270	257	1339
**Mean Age**	34.0 ± 6.6	33.1 ± 6.0**	34.7 ± 6.2	35.8 ± 5.6	35.0 ± 6.3	34.5 ± 6.3
**Weight (Kg)**	63.5 ± 13.1**	82.0 ± 22.3**	78.5 ± 18.6**	72.1 ± 17.3**	91.7 ± 24.4	77.2 ± 21.6
**Height (cm)**	158.0 ± 5.7**	160.1 ± 6.3**	163.2 ± 6.6	161.4 ± 6.5**	164.0 ± 6.2	161.2 ± 6.6
**BMI (Kg/m2)**	25.5 ± 5.2**	31.0 ± 8.2**	29.5 ± 6.7**	27.6 ± 6.2**	34.1 ± 8.8	29.6 ± 7.7
**Waist (cm)**	84.2 ± 12.5**	97.0 ± 16.4**	92.0 ± 13.8**	87.9 ± 12.3**	101.9 ± 19.6	92.3 ± 16.4
**Hip (cm)**	100.2 ± 13.3**	114.2 ± 15.7*	107.9 ± 11.6 **	104.4 ± 12.4**	117.1 ± 16.0	108.5 ± 15.2
**WHR**	0.84 ± 0.070**	0.85 ± 0.076**	0.85 ± 0.066**	0.84 ± 0.057**	0.87 ± 0.090	0.85 ± 0.073
**Overweight/Obese, (N) %**	141 (48.12)**	209 (77.99)*	181 (72.11)**	165 (61.11)**	219 (85.21)	915 (68.34)
**Smokers, (N) %**	0 (0.0)**	21 (7.87)**	43 (17.13)**	17 (6.3)**	84 (32.68)	165 (12.33)
**MVPA Duration (min/day)**	25.9 ± 16.7**	22.0 ± 16.2**	19.5 ± 15.3**	22.9 ± 14.7**	15.1 ± 17.7	21.2 ± 16.6

*p < 0.05, and **p < 0.01. Reference is USA

### Drinking patterns

Tables 3 and 4, and Supplementary Figs. 1 and 2 present the self-report drinking patterns for both men and women, respectively, stratified by site, among participants who indicated that they consumed alcohol. Across the five sites, about 19.4% of men qualified as abstainers, while 36.3% were heavy drinkers. Ghanaian men reported the lowest rates of drinking (42.5%), indicating they do not drink regularly. While only 9.4% of men from the USA did not drinking regularly. The highest rates of light drinking were observed among Jamaican men (67.1%), while the greatest rates were observed among South African men (19.5%). Seychellois men had the highest rates of heavy drinking (60.4%), while Ghanaian men reported the lowest rates of heavy drinking (8.7%). Overall, women were less likely to drink, with 46.0% of them reporting irregular drinking habit. Furthermore, 31.8% of women across all sites reported consuming 1–3 drinks daily on average, and 22.2% drank more than three alcoholic beverages each day. Ghanaian women had the lowest rates of drinking, with 77.5% indicating irregular drinking, while women from the Seychelles reported the highest rates of drinking, and only 14.8% indicated not drinking regularly. It was observed that women from the USA had the highest rates of light drinking (55.3%), while South Africans reported the lowest rates of light drinking among men (4.9%). Seychellois women had the highest rates of heavy drinking (45.9%), while Ghanaian women showed the lowest rates of heavy drinking (4.1%).

**Table 3:** Alcohol consumption data by site for men

	Ghana	South Africa	Jamaica	Seychelles	United States	Overall
**N**	207	236	249	230	245	1167
**Percent Drinkers (N) %**	119 (57.49)**	172 (72.88)**	222 (89.16)	206 (89.57)	222 (90.61)	941 (80.63)
**# Days Drink/yr**	148 ± 138**	145 ± 96**	90 ± 104	84 ± 97*	99 ± 102	108 ± 108
**# drinks/day**	1.9 ± 1.3**	6.5 ± 5.8**	2.7 ± 1.9*	5.2 ± 3.7**	3.9 ± 3.2	4.1 ± 3.9
**# drinks special occasion**	2.0 ± 2.2**	7.4 ± 7.1**	4.6 ± 4.6	10.0 ± 5.5**	4.8 ± 4.2	6.0 ± 5.7
**# Abstain, (N) %**	88 (42.51)**	64 (27.12)**	27 (10.84)	24 (10.43)	23 (9.39)	226 (19.37)
**# Light Drinker (1–3), (N) %**	101 (48.79)	46 (19.49)**	167 (67.07)*	67 (29.13)**	137 (55.92)	51 (44.39)
**# Heavy Drinker (> = 4), (N) %**	18 (8.70)**	126 (53.39)**	55 (22.09)**	139 (60.43)**	85 (34.69)	423 (36.25)

*p < 0.05, and **p < 0.01. Reference is USA. Categories for Abstain, Light drinker, and Heavy drinker are defined by the National Institute of Alcohol Abuse and Alcoholism (NIAAA). Number of Drinks a Day, Number of Drinking Days per year, and Number of Drinks on Special occasions exclude participants that indicated that they do not drink regularly

**Table 4:** Alcohol consumption data by site for women

	Ghana	South Africa	Jamaica	Seychelles	United States	Overall
**N**	293	268	251	270	257	1339
**Percent Drinkers (N) %**	66 (22.53)**	58 (21.64)**	157 (62.55)**	230 (85.19)	212 (82.49)	723 (54.00)
**# Days Drink/yr**	82 ± 117**	115 ± 92**	39 ± 68	39 ± 63	49 ± 70	51 ± 77
**# drinks/day**	1.4 ± 0.83**	5.5 ± 4.4**	2.1 ± 1.4*	3.1 ± 2.1*	2.6 ± 2.3	2.8 ± 2.4
**# drinks special occasion**	1.1 ± 1.5**	5.6 ± 4.8**	2.6 ± 2.7	4.8 ± 3.2**	3.1 ± 3.1	4.9 ± 0.24
**# Abstain (N) %**	227 (77.47)**	210 (78.36)**	94 (37.45)**	40 (14.81)	45 (17.51)	616 (46.00)
**# Light Drinker (1–2) (N) %**	54 (18.43)**	13 (4.85)**	111 (44.22)*	106 (39.26)**	142 (55.25)	426 (31.81)
**#Heavy Drinker (> = 3) (N) %**	12 (4.10)**	45 (16.79)**	46 (18.33)*	124 (45.93)**	70 (27.24)	297 (22.18)

*p < 0.05, and **p < 0.01. Reference is USA. Categories for Abstain, Light Drinker, and Heavy Drinker are defined by the National Institute of Alcohol Abuse and Alcoholism (NIAAA). Number of Drinks a Day, Number of Drinking Days per year, and Number of Drinks on Special occasions exclude participants that indicated that they do not drink regularly

### Cardiometabolic risk

The prevalence of CM risk is presented in Tables 5 and 6, and Supplementary Figs. 3 and 4, men, women, respectively. Overall, USA men had the highest prevalence of high CM risk (22.0%). However, Seychellois men had the highest number of participants with elevated blood glucose level (29.6%) (Supplementary Fig. 3). South African men had the greatest number of participants with high blood pressure (47.9%). Hypertriglyceridemia was observed more in Jamaican men (38.6%) and Ghanaian men had the highest number of participants with low HDL (44.4%). Abdominal obesity was observed among more men from the USA (35.0%).

**Table 5:** Cardiometabolic statistics and prevalence by site for men

	Ghana	South Africa	Jamaica	Seychelles	United States	Overall
**N**	207	236	249	230	245	1167
**Fasted blood glucose mg/dL**	100.9 ± 11.7	85.0 ± 13.8**	95.4 ± 9.1**	107.0 ± 37.3	105.8 ± 34.1	98.8 ± 25.7
**Elevated blood glucose (N) %**	41 (19.81)	12 (5.08)**	15 (6.02)**	68 (29.57)	61 (24.90)	197 (16.88)
**SBP (mmHg)**	118.9 ± 13.1**	129.0 ± 17.1**	121.5 ± 12.8*	122.7 ± 14.6**	127.8 ± 14.5	124.1 ± 15.0
**DBP (mmHg)**	68.5 ± 11.4**	79.6 ± 13.1**	71.2 ± 11.1**	75.0 ± 11.4**	81.0 ± 12.1	75.2 ± 12.7
**Elevated blood pressure, (N) %**	33 (15.94)**	113 (47.88)**	46 (18.47)**	79 (34.35)*	116 (47.35)	387 (33.16)
**Triglycerides (mg/dL)**	88.2 ± 45.9	105.2 ± 162.1	71.5 ± 32.8	97.0 ± 77.1	95.8 ± 57.0	93.0 ± 91.88
**Elevated triglycerides, (N) %**	25 (12.08)	36 (15.25)*	96 (38.55)**	36 (15.65)	38 (15.51)	231 (19.79)
**HDL (mg/dL)**	44.7 ± 16.4**	54.0 ± 19.8	47.1 ± 12.6**	47.4 ± 13.7**	50.1 ± 14.9	49.0 ± 16.4
**Low HDL, (N) %**	92 (44.44)**	62 (26.27)*	53 (21.29)	45 (19.57)	74 (30.20)	326 (27.93)
**LDL (mg/dL)**	92.5 ± 30.3*	80.5 ± 38.3**	94.5 ± 29.6**	112.8 ± 35.0	112.8 ± 34.2	97.6 ± 36.2
**Cholesterol (mg/dL)**	154.8 ± 35.3**	155.3 ± 40.3**	155.9 ± 32.8**	176.1 ± 35.9	182.0 ± 39.0	165.8 ± 38.9
**Ratio cholesterol/HDL (mg/dL)**	3.9 ± 12.2**	3.1 ± 1.1	3.5 ± 1.1	4.0 ± 1.2*	3.9 ± 1.2	3.6 ± 1.5
**CRP (mg/dL)**	3.8 ± 12.7	5.6 ± 11.6	2.2 ± 3.7	2.0 ± 2.2*	4.6 ± 11.6	3.7 ± 9.8
**Abdominal obesity, (N) %**	3 (1.46)	13 (5.63)	11 (4.42)**	29 (12.66)**	85 (34.98)	141 (12.19)
**Elevated CM risk, (N) %**	15 (7.25)	21 (8.90)	6 (2.41)*	35 (15.22)	54 (22.04)	131 (11.23)

*p < 0.05, and **p < 0.01. Reference is USA. Cardiometabolic conditions are defined using the ATP III Guidelines [30]

**Table 6:** Cardiometabolic statistics and prevalence by site for women

Women	Ghana	South Africa	Jamaica	Seychelles	United States	Overall
**N**	293	268	251	270	257	1339
**Fasted blood glucose mg/dL**	99.8 ± 12.5	83.1 ± 28.8**	90.9 ± 9.2**	95.3 ± 17.9	100.6 ± 35.4	94.0 ± 23.7
**Elevated blood sugar (N) %**	53 (18.09)	11 (4.10)**	9 (3.59)**	24 (8.89)*	44 (17.12)	141 (10.53)
**SBP (mmHg)**	110.4 ± 15.1**	118.1 ± 18.6	115.2 ± 14.7	110.9 ± 12.7**	117.4 ± 16.2	114.3 ± 15.9
**DBP (mmHg)**	66.1 ± 11.4**	76.3 ± 11.8	72.1 ± 11.4**	71.2 ± 9.9**	79.6 ± 13.2	72.9 ± 12.5
**Elevated blood pressure (N) %**	31 (10.58)**	192 (28.36)	52 (20.72)**	58 (21.48)**	89 (34.63)	306 (22.85)
**Trigs (mg/dL)**	77.5 ± 45.0**	76.3 ± 37.1**	74.3 ± 38.4**	64.5 ± 35.6**	96.7 ± 61.6	77.6 ± 43.5
**High triglycerides (N) %**	17 (5.8)**	11 (4.10)**	18 (7.17)**	12 (4.44)**	50 (19.46)	108 (8.07)
**HDL (mg/dL)**	47.1 ± 13.2**	46.2 ± 16.5**	46.1 ± 11.8**	47.9 ± 12.2**	51.8 ± 14.8	47.8 ± 14.1
**Low HDL (N) %**	156 (53.24)**	176 (65.67)**	156 (62.15)**	96 (35.56)	110 (42.80)	694 (51.83)
**LDL (mg/dL)**	103.4 ± 29.7	98.5 ± 27.8**	106.2 ± 28.4	109.3 ± 33.0	107.4 ± 33.1	104.6 ± 30.5
**Cholesterol (mg/dL)**	166.0 ± 34.4**	159.9 ± 33.3**	167.2 ± 33.9**	166 ± 34.5**	178.5 ± 38.6	167.3 ± 35.38
**Ratio cholesterol/HDL (mg/dL)**	3.7 ± 1.1**	3.7 ± 1.0	3.8 ± 0.98**	3.8 ± 1.2**	3.6 ± 0.97	3.7 ± 1.1
**CRP (mg/dL)**	5.3 ± 13.5	9.5 ± 19.6*	5.4 ± 7.2	3.9 ± 5.7	7.3 ± 9.7	6.3 ± 12.5
**Abdominal obesity (N) %**	95 (32.53)	179 (67.04)	148 (59.2)	124 (45.93)	196 (76.26)	742 (55.54)
**Elevated CM risk (N) %**	26 (8.87)**	42 (15.67)*	38 (15.14)*	28 (10.37)*	70 (27.24)	204 (15.24)

*p < 0.05, and **p < 0.01. Reference is USA. Cardiometabolic conditions are defined by using ATP III Guidelines [30]

More USA women (27.2%) had high CM risk (elevated blood pressure: 34.6%, hypertriglyceridemia: 19.5% and abdominal obesity: 76.3%) (Table 6, Supplementary Fig. 4). For the remaining individual risk factors, South African men had the highest rates of low HDL (65.7%), while women from Ghanaian and USA showed the highest prevalence for elevated blood sugar.

### Drinking and cardiometabolic risk

The results of the relationship between drinking and CM risk is presented in Table 7, and by individual site in Supplementary Tables 1, 2, 3, 4 and 5.

**Table 7:** Association of National Institute of Alcohol Abuse and Alcoholism drinking status and CM risk across all study sites

	ODDS RATIO	95% CONFIDENCE INTERVALS	***P***-value
**Elevated blood glucose **			
**Light drinkers**	0.760	0.543↔1.06	0.110
**Heavy drinkers**	0.890	0.610↔1.30	0.548
**Elevated blood pressure**			
**Light drinkers**	1.14	0.871↔1.49	0.343
**Heavy drinkers**	1.59	1.20↔2.10	0.001
**High triglycerides**			
**Light drinkers**	1.48	1.04↔2.12	0.030
**Heavy drinkers**	1.73	1.17↔2.55	0.006
**Low HDL**			
**Light drinkers**	0.690	0.548↔0.868	0.002
**Heavy drinkers**	0.621	0.480↔0.803	< 0.0005
**Abdominal obesity**			
**Light drinkers**	0.829	0.626↔1.10	0.193
**Heavy drinkers**	0.961	0.707↔1.31	0.803
**High CM risk**			
**Light drinkers**	1.05	0.745↔1.47	0.792
**Heavy drinkers**	1.11	0.787↔1.64	0.489

Associations adjusted for site, sex, smoking status, physical activity, and age. The reference is non-drinking status as defined by the National Institute of Alcohol Abuse and Alcoholism (NIAAA) Guidelines [29]

### Elevated Blood pressure

Overall, heavy drinkers had significantly increased odds for high BP when compared to non-drinkers (odds ratio (OR) = 1.59, *p* = 0.001). Men who were heavy drinkers had significantly higher odds of elevated BP in comparison to men who were non-drinkers (OR = 2.25, *p* < 0.001). However, this association was not significant among men who were light drinkers when compared to men who were non-drinkers (OR = 1.43, *p* = 0.089). Women did not show any significant associations for alcohol consumption and high BP.

Light drinkers had 2.05 times the odds of having high BP compared to non-drinkers (*p* = 0.022). Heavy drinkers had 2.93 times the odds of having hypertension, compared to non-drinkers (*p* = 0.002); however, this association was primarily driven by USA men (OR = 3.5, *p* = 0.016). Both light and heavy drinking status among USA women showed tendencies towards higher odds of elevated BP, although neither of these associations showed significance (light drinkers: OR = 2.16, *p* = 0.061; heavy drinkers: OR = 2.31, *p* = 0.072).

### Hypertriglyceridemia

Overall, both light and heavy drinking were significantly associated with an increased odds of hypertriglyceridemia. Light drinking participants had 1.48 times the odds of having high triglyceride levels compared to non-drinkers (*p* = 0.030), while heavy drinkers had 1.73 times the odds of having hypertriglyceridemia compared to non-drinkers (*p* = 0.006). Only Ghanaians had associations between drinking status and triglyceride levels, where both light and heavy drinking were significantly associated with hypertriglyceridemia.

### Low HDL

Both light and heavy drinking were significantly associated with having low HDL. Light drinkers had 0.69 times the odds of having low HDL compared to non-drinkers (*p* = 0.002), while heavy drinking participants had 0.621 times the odds of having low HDL compared to non-drinkers (*p* < 0.0005). Overall, men were more likely to have a significant association between heavy drinking and low HDL (OR = 0.516, *p* = 0.002). Similarly light drinking females were more likely to have healthy HDL levels (OR = 0.677, *p* = 0.013).

For the site-specific associations, heavy drinking Ghanaians were significantly more likely to have low HDL (OR = 3.51, *p* = 0.021) (Supplementary Table 1). Among South Africans, light drinkers had 0.359 times the odds for having low HDL compared to non-drinkers (*p* = 0.004) and heavy drinking participants had 0.314 times the odds for having low HDL compared to nondrinkers (*p* < 0.0005) (Supplementary Table 2). These associations remained significant after stratifying by sex. Light drinking men had 0.319 times the odds (*p* = 0.015) and heavy drinking men had 0.281 the odds of low HDL (*p* = 0.001) when compared to non-drinking counterparts. Among South African women, drinking was associated with low HDL (light drinkers: OR = 0.287, *p* = 0.035; heavy drinkers OR = 0.314, *p* = 0.004). Among subjects from the USA, heavy drinking was significantly associated with having a decreased odds for low HDL (OR = 0.513, *p* = 0.040).

### Waist circumference

The study could not account for significant associations between levels of alcohol consumption and abdominal obesity. Site-specific associations showed that among Ghanaians, light drinking was significantly associated with having abdominal obesity (OR = 2.15, *p* = 0.017). This association was driven by Ghanaian women, among who light drinking participants had 2.15 times the odds of having abdominal obesity compared to nondrinkers (*p* = 0.015). Similarly, women who were heavy drinkers from the USA had a strong trend of having abdominal obesity, but this was not statistically significant (OR = 2.44, *p* = 0.090).

### Cardiometabolic risk

Overall, we did not find a significant association between alcohol consumption status and elevated CM risk (3 out of 5 risk factors). However, among heavy drinking males, there was a strong trend for elevated CM risk (OR = 1.87, *p* = 0.065). In addition, only Ghanaians showed relationships between alcohol consumption and elevated CM risk. Light drinking Ghanaians had an increased odds for having elevated CM risk (OR = 2.15, *p* = 0.017), while heavy drinking Ghanaian men were significantly more likely to present with elevated CM risk (OR = 10.4, *p* = 0.012). Similarly, light drinking Ghanaian women were significantly more likely to present with elevated CM risk (OR = 2.48, *p* = 0.046).

## Discussion

We evaluated the association between alcohol consumption and elevated CM risk in five African-origin populations spanning the epidemiologic transition. The study found that among the groups, heavy alcohol consumption tended to be associated with an increased odds for presenting with elevated CM risk. It was observed that within sites, drinking status was associated with the various components of CM risk, including elevated BP, high triglycerides and low HDL. These associations varied greatly across each of the five sites, suggesting an effect of environmental factors on CM risk. These relationships also varied when stratified by sex indicating that sex may modify the association between alcohol consumption and CM risk.

We did, however, find that the association between alcohol consumption and increased CM risk is not consistent. For example, Freiberg et al. found that moderate alcohol consumption was significantly with a decreased risk for elevated CM risk [22]. This is in contrast to a recent meta-analysis from the Global Burden of Disease Study group, which suggested that alcohol consumption significantly increased CM risk [10]. Each of these studies, regardless of findings, demonstrated a dose-response effect, in which increased alcohol consumption resulted in either the protective or increased risk for the five CM risk factors [10, 21, 22, 31, 32]. While our study found no significant association between any amount of alcohol consumption and having increased CM risk, the data suggest a trend between heavy alcohol consumption and risk for CM among the men. This association was however, negated among the women as well as for the site-specific analyses, except among Ghanaian light drinkers who had a significant odds for having elevated CM risk. It is important to note that the lack of a significant association between heavy drinking and elevated CM risk in Ghana may be due to the small number of heavy drinkers, as only eighteen Ghanaian men and twelve Ghanaian women qualified as heavy drinkers.

While previous studies have not investigated the association between alcohol consumption and high CM risk specifically among African-origin populations, several studies have explored the prevalence of high CM risk in each of our five cohorts. For example, the prevalence of high CM risk in Ghana was found to be 5.9% in men and 24% in women in 2012 [33]. In South Africa, the prevalence was 10.5% in men and 25% in women in 2011 and 11% of men and 23% of women in Jamaica had high CM risk in 2008 [18]. In Seychelles, the prevalence was 24% in men and 32.2% in women in 2008 [27]; and among US-African Americans, 24% of men and 42% of women had the metabolic disorder in 2003. With the exception of Ghanaian men, these previous studies reported higher rates of high CM risk than this investigation. In some cases, the differences in prevalence exceeded 20%, as was the case for the USA and Seychelles.

However, our data, also, identified similar findings as previous studies. For example, we similarly identified higher prevalence of CM risk among Ghanaian women compared to Ghanaian men. Similarly, we identified a higher prevalence of low HDL among Ghanaian and South African women when compared to Ghanaian and South African men, respectively. Our data and previous data showed that prevalence of having an elevated CM risk increased with the site’s developmental index, i.e. lowest prevalence among Ghanaians and highest prevalence among the US cohort. Similar to previous literature, we confirmed that women had higher rates of elevated CM risk than men [18–20, 34]. Similarities to previous research are limited as, to our knowledge, no other study has investigated the association between alcohol intake and CM risk in these populations.

Alcohol consumption has also previously been measured in each of the five cohorts. For example, previous studies have found that approximately 80% of Ghanaians were regular drinkers [34], 56% of men and 26% of women from South Africa admitted to drinking regularly [16] and in Jamaica 57% of men and 35% of women reported that they were regular drinkers [16]. In the Seychelles, approximately 51% of men and 6% of women were regular drinkers [17]; and about 75.5% of men and 63% of women reported to be drinking regularly in the USA [16]. These studies are not in line with our study, and may be partly due to the different classification of alcohol consumption, as well as differences in the methods and questions used to obtain self-reported data. Self-reported data generally introduce variation as the participants subjectively provide these data as opposed to objectively measuring alcohol consumption. Therefore, the methods used to obtain these self-reported data may introduce variation between studies. Despite these differences, we did find that more women were more likely to abstain from alcohol consumption compared to men in all sites.

One of the primary limitations of this study is that all alcohol data were self-reported, thus introducing biases, such as social desirability. Depending on the country of origin and social tolerance to alcohol drinking, some participants may under-report their alcohol consumption, and such systematic misclassification may be related to sex or different population sub-groups. Furthermore, alcohol habits may change over time, including low consumption or abstinence; following disease, itself possibly related to alcohol. However, this is not likely the case due to the relatively young ages of the study participants. Another limitation is that alcohol drinking patterns (e.g. binge vs. regular) and alcohol types (e.g. wine, beer, spirts, home-brews), which are factors that may alter the impact alcohol intake has on CM risk, were not captured. The prevalence of heavy drinking was relatively low in most of the sites, particularly in women, which minimizes statistical power for association analysis. In some instances, we were underpowered to detect statistical significance. For example, we were unable to test the association between heavy drinking status and risk for elevated blood glucose in Ghanaian women, as none of the heavy drinking female Ghanaians had hyperglycemia. Indeed, this site confounding may have masked some of the associations, although we attempted to address this by using dummy variables for site. Finally, it is a major limitation that we were unable to describe any potential mechanisms for the association between alcohol intake and prevalence of CM risk in our international cohort. However, our study is strengthened by the objective clinical measures, performed by certified staff, using standardized procedures and the same make of equipment across the five sites. Additionally, the biochemical samples were centrally stored and analyzed at the same laboratory.

## Conclusion

While we were able to detect several statistically significant associations between individual CM risk factors and alcohol consumption, these associations were inconsistent. The associations between alcohol consumption and elevated CM risk and its components varied markedly between the 5 cohorts in this study. This is not unexpected, given the current widely reported alcohol consumption effects. Alcohol intake was indeed, inversely related to lower HDL and higher triglycerides in nearly all sites in our study. While cost-effective strategies to reduce alcohol consumption have been implemented, our data also underscore differences on health outcomes between countries, and the need to tailor national programs and policies accordingly. Future studies should focus on understanding the individual site-related effects.

## Data Availability

The datasets used and/or analyzed during the current study are available from the corresponding author on reasonable request.

## Addtional files

**Additional file 1: Supplementary Fig. 1.** Mean Daily Alcohol Consumption for Men, by Site. Reference is USA. Excludes participants that indicated that they do not drink regularly. **p* < 0.05, and ***p* < 0.01. **Additional file 2: Supplementary Fig. 2.** Mean Daily Alcohol Consumption for Women, by Site. Reference is USA. Excludes participants that indicated that they do not drink regularly. **p* < 0.05, and ***p* < 0.01. **Additional file 3: Supplementary Fig. 3.** Prevalence of Cardiometabolic risk for Men, by Site. Reference is USA. **p* < 0.05, and ***p* < 0.01. **Additional file 4: Supplementary Fig. 4.** Prevalence of Cardiometabolic for Women, by Site. Reference is USA. **p* < 0.05, and ***p* < 0.01. **Additional file 5: Supplementary Table 1.** Association of National Institute of Alcohol Abuse and Alcoholism drinking status and CM risk in Ghana. **Supplementary Table 2.** Association of National Institute of Alcohol Abuse and Alcoholism drinking status and CM risk in South Africa. **Supplementary Table 3.** Association of National Institute of Alcohol Abuse and Alcoholism drinking status and CM risk in Jamaica. **Supplementary Table 4.** Association of National Institute of Alcohol Abuse and Alcoholism drinking status and CM risk in Seychelles. **Supplementary Table 5.** Association of National Institute of Alcohol Abuse and Alcoholism drinking status and CM risk in USA.

## Abbreviations

BP: Blood Pressure
CM: Cardiometabolic
HDI: Human Development Index
HDL: High-Density Lipoprotein
METS: Modeling the Epidemiologic Transition Study
NIAAA: National Institute of Alcohol Abuse and Alcoholism
OR: Odds Ratio
TG: Triglycerides
WHO: World Health Organization
