# Supplementary figures and images for: The associations between alcohol intake and cardiometabolic risk in African-origin adults spanning the epidemiologic transition
Source: BMC Public Health. 2021 Dec 4;21:2210. doi: 10.1186/s12889-021-12128-2 (PMC8642964; doi:10.1186/s12889-021-12128-2)

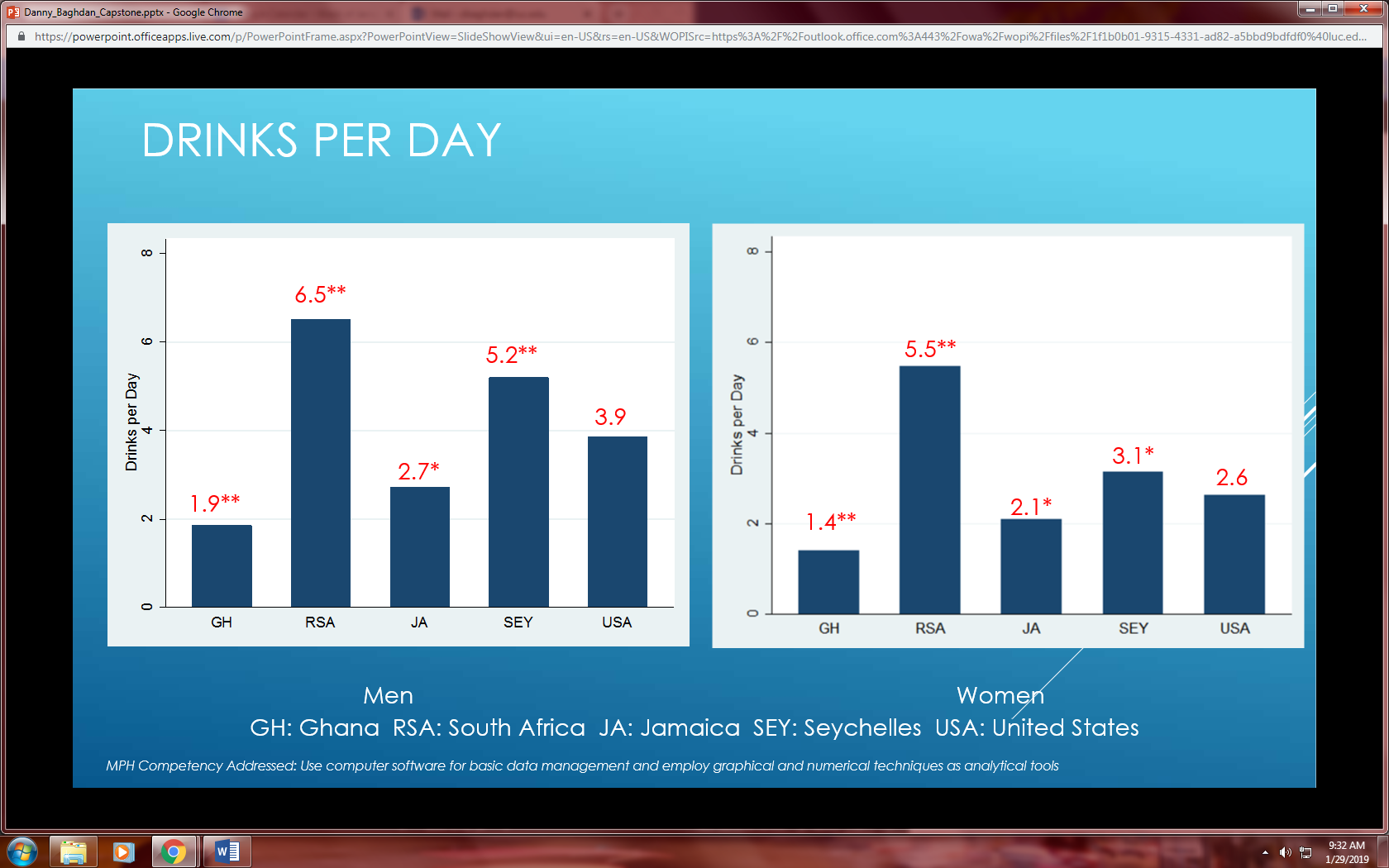

Supplement: Supplementary file 1 — Additional file 1: Supplementary Fig. 1. Mean Daily Alcohol Consumption for Men, by Site. Reference is USA. Excludes participants that indicated that they do not drink regularly. *p < 0.05, and **p < 0.01. [file 12889_2021_12128_MOESM1_ESM.docx]

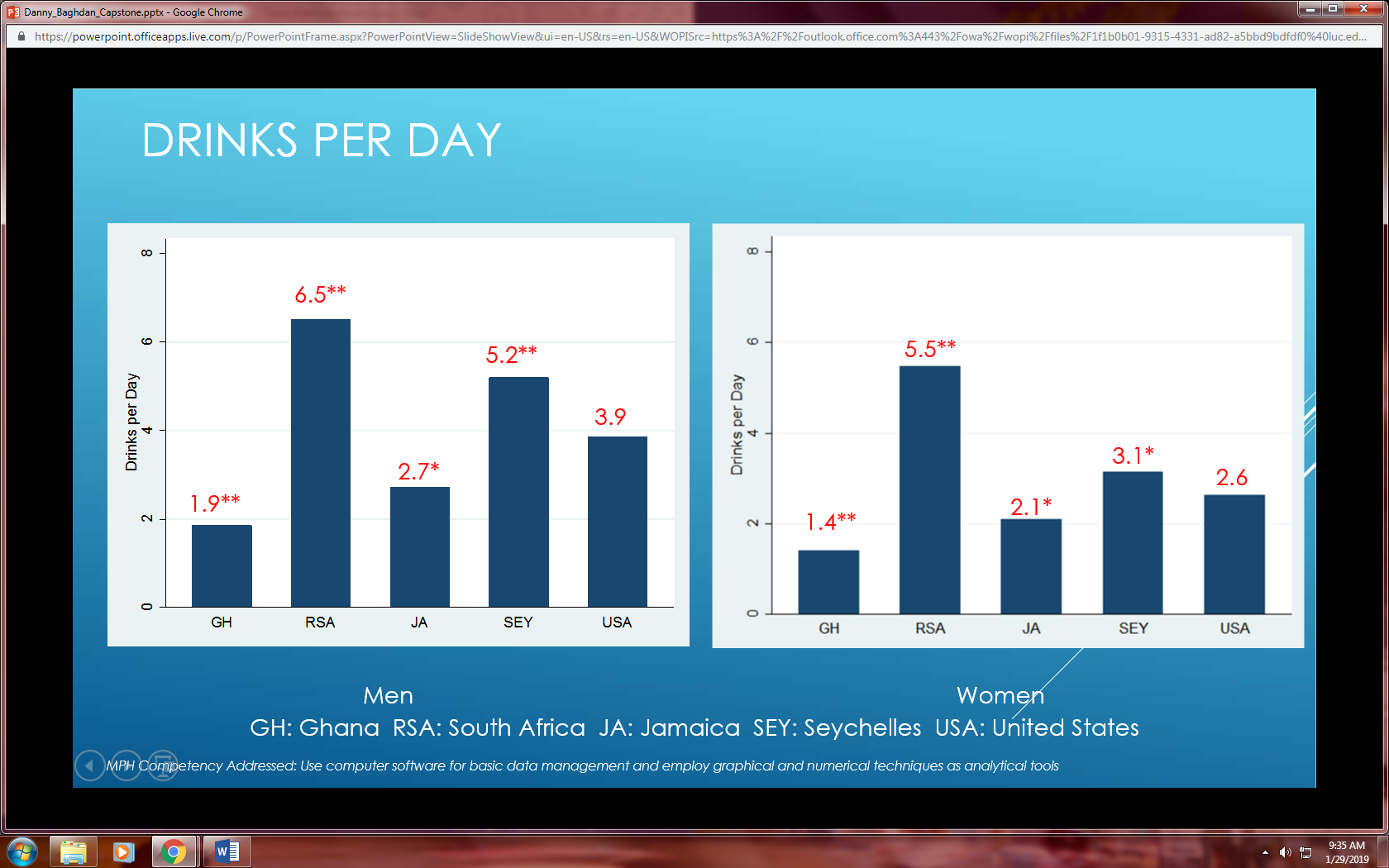

Supplement: Supplementary file 2 — Additional file 2: Supplementary Fig. 2. Mean Daily Alcohol Consumption for Women, by Site. Reference is USA. Excludes participants that indicated that they do not drink regularly. *p < 0.05, and **p < 0.01. [file 12889_2021_12128_MOESM2_ESM.docx]

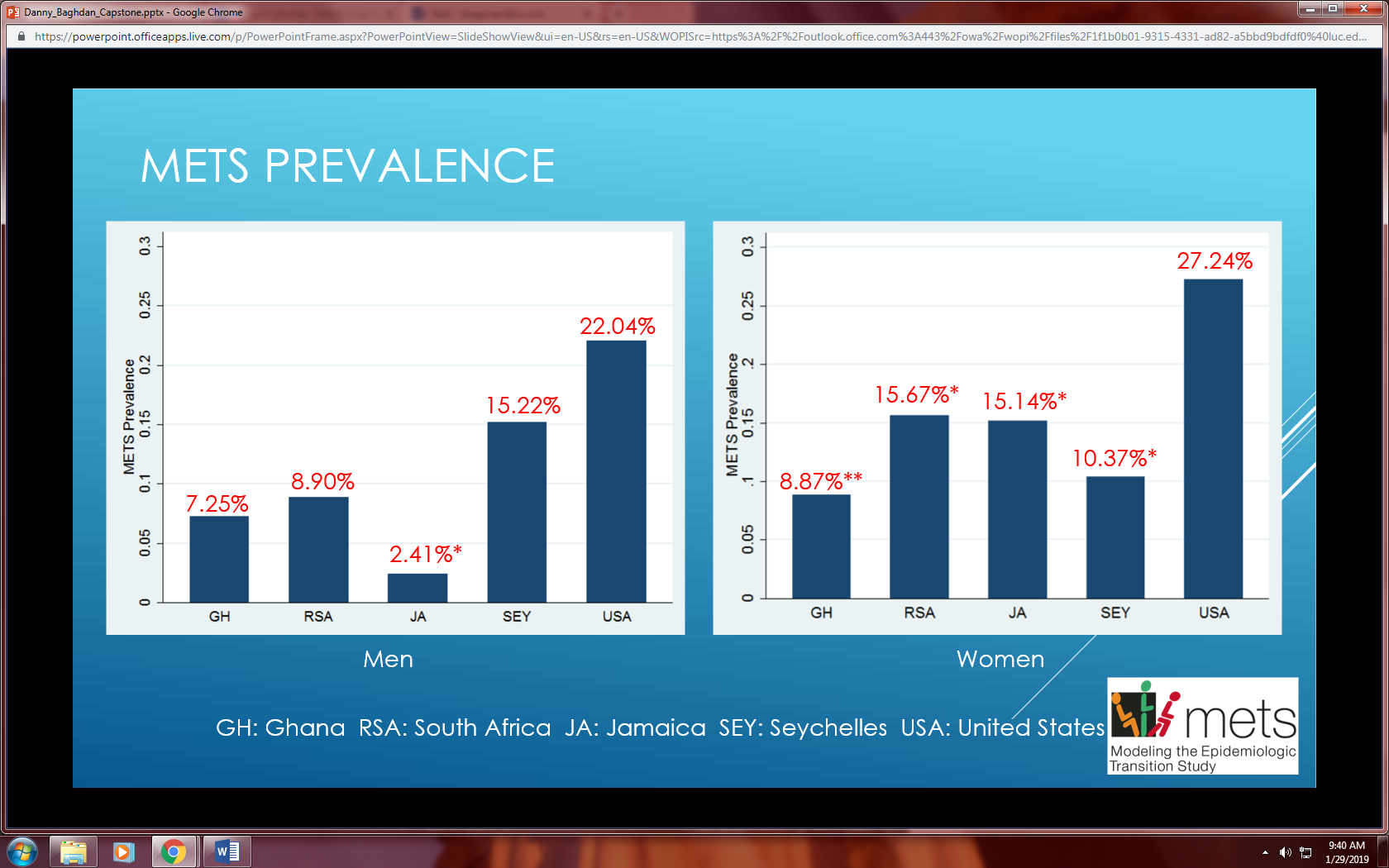

Supplement: Supplementary file 3 — Additional file 3: Supplementary Fig. 3. Prevalence of Cardiometabolic risk for Men, by Site. Reference is USA. *p < 0.05, and **p < 0.01. [file 12889_2021_12128_MOESM3_ESM.docx]

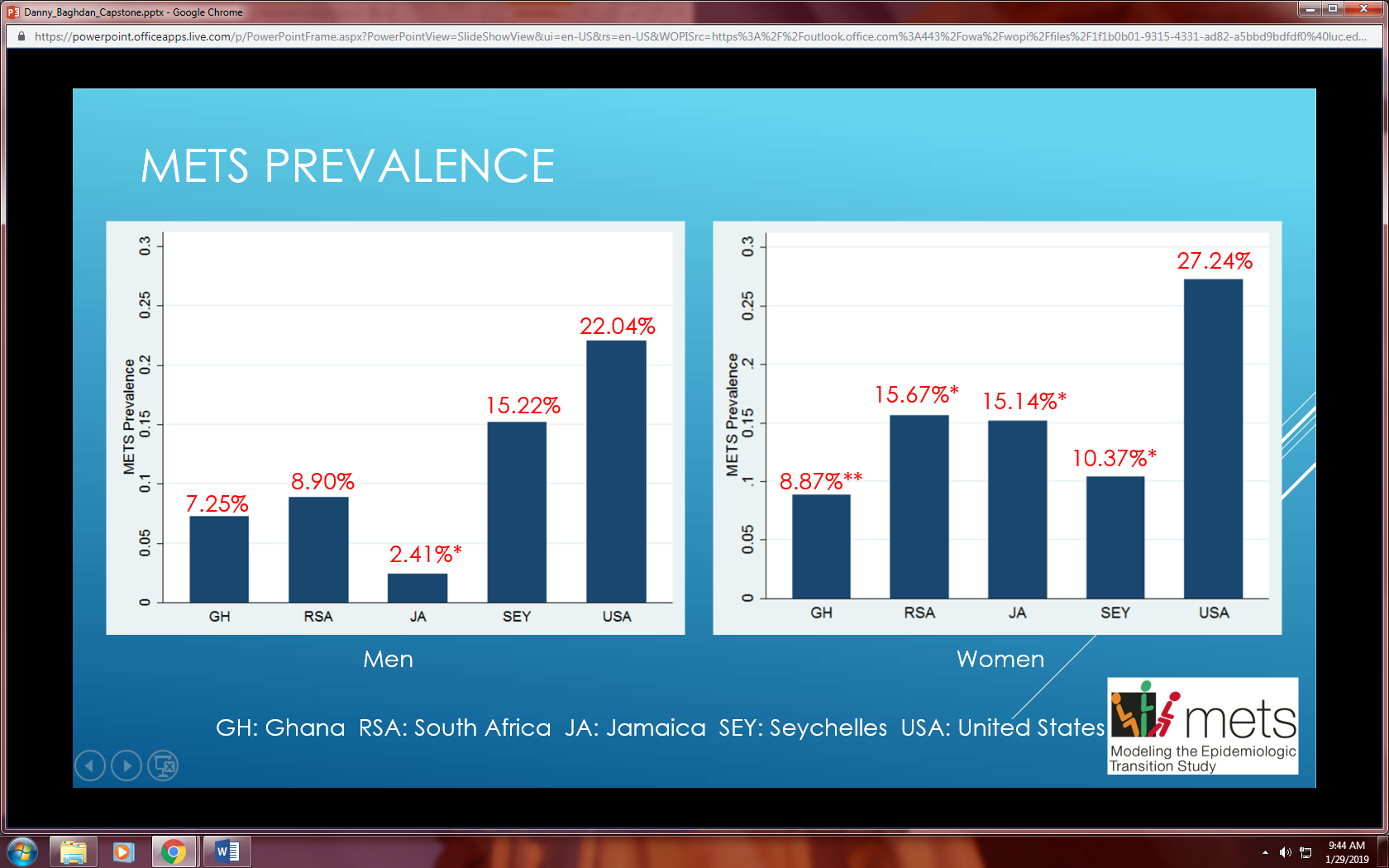

Supplement: Supplementary file 4 — Additional file 4: Supplementary Fig. 4. Prevalence of Cardiometabolic for Women, by Site. Reference is USA. *p < 0.05, and **p < 0.01. [file 12889_2021_12128_MOESM4_ESM.docx]
