# Supplementary material for: The associations between alcohol intake and cardiometabolic risk in African-origin adults spanning the epidemiologic transition
Source: BMC Public Health. 2021 Dec 4;21:2210. doi: 10.1186/s12889-021-12128-2 (PMC8642964; doi:10.1186/s12889-021-12128-2)
Supplement: Supplementary file 5 — Additional file 5: Supplementary Table 1. Association of National Institute of Alcohol Abuse and Alcoholism drinking status and CM risk in Ghana. Supplementary Table 2. Association of National Institute of Alcohol Abuse and Alcoholism drinking status and CM risk in South Africa. Supplementary Table 3. Association of National Institute of Alcohol Abuse and Alcoholism drinking status and CM risk in Jamaica. Supplementary Table 4. Association of National Institute of Alcohol Abuse and Alcoholism drinking status and CM risk in Seychelles. Supplementary Table 5. Association of National Institute of Alcohol Abuse and Alcoholism drinking status and CM risk in USA. [file 12889_2021_12128_MOESM5_ESM.docx]

**Supplementary Table 1: Association of National Institute of Alcohol Abuse and Alcoholism drinking status and CM risk in Ghana**

|  |  | **ODDS RATIO** | **95% CONFIDENCE INTERVALS** | **P-value** |
| --- | --- | --- | --- | --- |
| **Elevated Blood Sugar** | | | | |
|  | **Light drinkers** | 1.08 | 0.626↔1.85 | 0.789 |
|  | **Heavy drinkers** | 0.851 | 0.270↔2.68 | 0.782 |
| **Elevated Blood Pressure** | | | | |
|  | **Light drinkers** | 2.29 | 1.25↔4.22 | 0.008 |
|  | **Heavy drinkers** | 1.09 | 0.289↔4.12 | 0.898 |
| **High Triglycerides** | | | | |
|  | **Light drinkers** | 2.30 | 1.06↔5.00 | 0.035 |
|  | **Heavy drinkers** | 6.39 | 2.07↔19.7 | 0.001 |
| **Low HDL** | | | | |
|  | **Light drinkers** | 0.740 | 0.477↔1.15 | 0.178 |
|  | **Heavy drinkers** | 3.51 | 1.21↔10.2 | 0.021 |
| **Abdominal Obesity** | | | | |
|  | **Light drinkers** | 2.15 | 1.14↔4.05 | 0.017 |
|  | **Heavy drinkers** | 1.91 | 0.565↔6.46 | 0.297 |
| **High CM Risk** | | | | |
|  | **Light drinkers** | 2.25 | 1.08↔4.68 | 0.030 |
|  | **Heavy drinkers** | 2.19 | 0.577↔8.35 | 0.249 |

Associations adjusted for sex, smoking status, physical activity, and age. The reference is Non-Drinking status as defined by the National Institute of Alcohol Abuse and Alcoholism (NIAAA) Guidelines (23).

**Supplementary Table 2: Association of National Institute of Alcohol Abuse and Alcoholism drinking status and CM risk in South Africa**

|  |  | **ODDS RATIO** | **95% CONFIDENCE INTERVALS** | **P-value** |
| --- | --- | --- | --- | --- |
| **Elevated Blood Sugar** | | | | |
|  | **Light drinkers** | 1.17 | 0.307↔4.44 | 0.819 |
|  | **Heavy drinkers** | 0.520 | 0.155↔1.75 | 0.290 |
| **Elevated Blood Pressure** | | | | |
|  | **Light drinkers** | 1.28 | 0.659↔2.50 | 0.464 |
|  | **Heavy drinkers** | 1.79 | 1.11↔2.91 | 0.018 |
| **High Triglycerides** | | | | |
|  | **Light drinkers** | 1.06 | 0.375↔3.01 | 0.908 |
|  | **Heavy drinkers** | 1.24 | 0.562↔2.73 | 0.595 |
| **Low HDL** | | | | |
|  | **Light drinkers** | 0.359 | 0.179↔0.720 | 0.004 |
|  | **Heavy drinkers** | 0.314 | 0.191↔0.515 | <0.0005 |
| **Abdominal Obesity** | | | | |
|  | **Light drinkers** | 0.753 | 0.272↔2.08 | 0.584 |
|  | **Heavy drinkers** | 0.962 | 0.483↔1.91 | 0.912 |
| **High CM Risk** | | | | |
|  | **Light drinkers** | 1.04 | 0.372↔2.91 | 0.940 |
|  | **Heavy drinkers** | 0.854 | 0.334↔1.84 | 0.687 |

Associations adjusted for sex, smoking status, physical activity, and age. The reference is Non-Drinking status as defined by the National Institute of Alcohol Abuse and Alcoholism (NIAAA) guidelines (23).

**Supplementary Table 3: Association of National Institute of Alcohol Abuse and Alcoholism drinking status and CM risk in Jamaica**

|  |  | **ODDS RATIO** | **95% CONFIDENCE INTERVALS** | **P-value** |
| --- | --- | --- | --- | --- |
| **Elevated Blood Sugar** | | | | |
|  | **Light drinkers** | 0.962 | 0.281↔3.30 | 0.951 |
|  | **Heavy drinkers** | 1.43 | 0.371↔5.49 | 0.605 |
| **Elevated Blood Pressure** | | | | |
|  | **Light drinkers** | 0.567 | 0.319↔1.01 | 0.054 |
|  | **Heavy drinkers** | 0.677 | 0.331↔1.39 | 0.286 |
| **High Triglycerides** | | | | |
|  | **Light drinkers** | 1.44 | 0.713↔2.90 | 0.310 |
|  | **Heavy drinkers** | 1.10 | 0.475↔2.56 | 0.820 |
| **Low HDL** | | | | |
|  | **Light drinkers** | 1.08 | 0.646↔1.82 | 0.760 |
|  | **Heavy drinkers** | 0.868 | 0.456↔1.65 | 0.665 |
| **Abdominal Obesity** | | | | |
|  | **Light drinkers** | 0.747 | 0.419↔1.33 | 0.321 |
|  | **Heavy drinkers** | 1.18 | 0.549↔2.54 | 0.669 |
| **High CM risk** | | | | |
|  | **Light drinkers** | 0.971 | 0.449↔2.10 | 0.939 |
|  | **Heavy drinkers** | 1.09 | 0.403↔2.95 | 0.864 |

Associations adjusted for sex, smoking status, physical activity, and age. The reference is Non-Drinking status as defined by the National Institute of Alcohol Abuse and Alcoholism (NIAAA) Guidelines (23).

**Supplementary Table 4: Association of National Institute of Alcohol Abuse and Alcoholism drinking status and CM risk in Seychelles**

|  |  | **ODDS RATIO** | **95% CONFIDENCE INTERVALS** | **P-value** |
| --- | --- | --- | --- | --- |
| **Elevated Blood Sugar** | | | | |
|  | **Light drinkers** | 0.536 | 0.227↔1.27 | 0.156 |
|  | **Heavy drinkers** | 0.725 | 0.324↔1.62 | 0.433 |
| **Elevated Blood Pressure** | | | | |
|  | **Light drinkers** | 0.521 | 0.255↔1.06 | 0.074 |
|  | **Heavy drinkers** | 0.934 | 0.479↔1.82 | 0.841 |
| **High Triglycerides** | | | | |
|  | **Light drinkers** | 0.800 | 0.196↔3.26 | 0.756 |
|  | **Heavy drinkers** | 1.78 | 0.496↔6.40 | 0.376 |
| **Low HDL** | | | | |
|  | **Light drinkers** | 1.09 | 0.548↔2.15 | 0.814 |
|  | **Heavy drinkers** | 1.11 | 0.571↔2.17 | 0.751 |
| **Abdominal Obesity** | | | | |
|  | **Light drinkers** | 0.683 | 0.349↔1.34 | 0.266 |
|  | **Heavy drinkers** | 0.619 | 0.320↔1.19 | 0.153 |
| **High CM risk** | | | | |
|  | **Light drinkers** | 0.532 | 0.201↔1.41 | 0.204 |
|  | **Heavy drinkers** | 0.883 | 0.363↔2.15 | 0.785 |

Associations adjusted for sex, smoking status, physical activity and age. The reference is non-drinking status as defined by the National Institute of Alcohol Abuse and Alcoholism (NIAAA) Guidelines (23).

**Supplementary Table 5: Association of National Institute of Alcohol Abuse and Alcoholism drinking status and CM risk in USA**

|  |  | **ODDS RATIO** | **95% CONFIDENCE INTERVALS** | **P-value** |
| --- | --- | --- | --- | --- |
| **Elevated Blood Sugar** | | | | |
|  | **Light drinkers** | 0.998 | 0.497↔2.00 | 0.996 |
|  | **Heavy drinkers** | 1.26 | 0.589↔0.68 | 0.555 |
| **Elevated Blood Pressure** | | | | |
|  | **Light drinkers** | 2.05 | 1.11↔3.78 | 0.022 |
|  | **Heavy drinkers** | 2.93 | 1.50↔5.74 | 0.002 |
| **High Triglycerides** | | | | |
|  | **Light drinkers** | 0.960 | 0.465↔1.98 | 0.912 |
|  | **Heavy drinkers** | 1.11 | 0.499↔2.46 | 0.802 |
| **Low HDL** | | | | |
|  | **Light drinkers** | 0.613 | 0.348↔1.08 | 0.090 |
|  | **Heavy drinkers** | 0.513 | 0.271↔0.970 | 0.040 |
| **Abdominal Obesity** | | | | |
|  | **Light drinkers** | 1.18 | 0.622↔2.24 | 0.610 |
|  | **Heavy drinkers** | 1.69 | 0.828↔3.43 | 0.150 |
| **High CM risk** | | | | |
|  | **Light drinkers** | 1.42 | 0.737↔2.74 | 0.294 |
|  | **Heavy drinkers** | 1.38 | 0.663↔2.88 | 0.387 |

Associations adjusted for sex, smoking status, physical activity, and age. The reference is non-drinking status as defined by the National Institute of Alcohol Abuse and Alcoholism (NIAAA) Guidelines (23).
